# Characterizing Hydropathy of Amino Acid Side Chain in a Protein Environment by Investigating the Structural Changes of Water Molecules Network

**DOI:** 10.3389/fmolb.2021.626837

**Published:** 2021-02-26

**Authors:** Lorenzo Di Rienzo, Mattia Miotto, Leonardo Bò, Giancarlo Ruocco, Domenico Raimondo, Edoardo Milanetti

**Affiliations:** ^1^ Center for Life Nanoscience, Istituto Italiano di Tecnologia, Rome, Italy; ^2^ Department of Physics, Sapienza University, Rome, Italy; ^3^ Department of Molecular Medicine, Sapienza University, Rome, Italy

**Keywords:** hydropathy, molecular dynamics simulation, hydrophobicity, local structural environment, water molecules network

## Abstract

Assessing the hydropathy properties of molecules, like proteins and chemical compounds, has a crucial role in many fields of computational biology, such as drug design, biomolecular interaction, and folding prediction. Over the past decades, many descriptors were devised to evaluate the hydrophobicity of side chains. In this field, recently we likewise have developed a computational method, based on molecular dynamics data, for the investigation of the hydrophilicity and hydrophobicity features of the 20 natural amino acids, analyzing the changes occurring in the hydrogen bond network of water molecules surrounding each given compound. The local environment of each residue is complex and depends on the chemical nature of the side chain and the location in the protein. Here, we characterize the solvation properties of each amino acid side chain in the protein environment by considering its spatial reorganization in the protein local structure, so that the computational evaluation of differences in terms of hydropathy profiles in different structural and dynamical conditions can be brought to bear. A set of atomistic molecular dynamics simulations have been used to characterize the dynamic hydrogen bond network at the interface between protein and solvent, from which we map out the local hydrophobicity and hydrophilicity of amino acid residues.

## 1 Introduction

Hydration water molecules play a crucial role in living organisms as most biological processes occur in an aqueous environment (Rothschild and Mancinelli, 2001), which actively influences the structure and function of biomolecules and their interactions (Levy and Onuchic, 2006; Ball 2008). Compounds immersed in water display different behaviors depending on their chemical characteristics. In particular, the arrangement of the water molecules that hydrate compounds changes according to their properties (Vagenende and Trout, 2012; Tomobe et al., 2017). So we can extract information on the chemical nature and function of the solute by studying the attraction and repulsion of chemical compounds toward the water (Chothia, 1976). In general, both hydrophobic and hydrophilic effects are dominant driving forces for several biochemical processes, such as protein folding, nucleic acid stability, molecular recognition, and binding (Tanford, 1972; Brooks et al., 1998; Aftabuddin and Kundu, 2007; Moret and Zebende, 2007; Miotto et al., 2018).

In light of this, solvation water should be considered an integral part of biological macromolecules. In particular, water molecules in solutions are divided into 1) internal water molecules that occupy cavities in the biomolecule structure and can be identified in crystallography; 2) water molecules that interact with the molecular surface and 3) bulk water. Depending on the category, the organization of the water molecules is associated with different time scales. The relaxation times for internal waters range from tens of ns to ms since they require local rearrangement of the protein to occur. On the other hand, the motion of bulk water has the time scale of the picoseconds. In between, there is the motion of surface water molecules that are characterized by residence times on the order of tens of picoseconds (Tarek and Tobias, 2000; Qvist et al., 2009; Mondal et al., 2017).

In general, the investigation of the behavior of water in the hydration shells of organic compounds is a fundamental analysis to better understand most biological processes both from a theoretical and practical point of view (Raschke, 2006).

An effective measure of the interaction between water and amino acids, the hydropathy index (a number representing the hydrophobic or hydrophilic properties of its side chain), was firstly proposed in 1982 by Kyte *et al.* (Kyte and Doolittle, 1982). Indeed, in the computational biology field, attributing a single number, the hydropathy index, to each amino acid is very useful for studying the chemical-physical and structural properties of proteins. Over the past few decades, many hydrophobicity and hydrophilicity scales, based on both experimental and theoretical approaches, have been defined, and these schematizations have proven their usefulness in the characterization of protein regions and the development of computational methods (Chothia, 1974; Jones, 1975; Kyte and Doolittle, 1982; Sweet and Eisenberg, 1983; Rose et al., 1985; Wilce et al., 1995). For instance, one of the typical use of the hydrophobicity and hydrophilicity values for the 20 amino acids is the prediction of transmembrane regions in protein structure modeling (Deber et al., 2001).

Recently we have developed a new theoretical-computational method analyzing the orientation of water molecules surrounding a small organic compound, as computed from molecular dynamics simulations (Bonella et al., 2014). The procedure is based on the calculation of the conditional probability density of finding a water molecule with a specific orientation, given its distance from the nearest atom of the solute (Babiaczyk et al., 2010; Bonella et al., 2014).

We thus applied this method to the 20 natural amino acids defining the *WOPHS* (Water Orientation Probability Hydropathy Scale) hydropathy scale, the first scale to be *vectorial* as it associates three indices for each amino acid (Bonella et al., 2014). In fact, we argued that assigning a single number is not enough to characterize the solvation properties of amino acids, in particular when both hydrophobic and hydrophilic regions are present in the same residue. In this respect, our characterization can be used to understand some of the known ambiguities in the ranking of amino acids in the current scales available in the literature. This method presents several advantages over previously developed computational and experimental approaches: it is sensitive to the specific environment of the amino acids and can be applied to unnatural and modified amino acids, as well as to other small organic molecules (Bonella et al., 2014; Leopizzi et al., 2017). In particular, analyzing the structural changes of the dynamic hydrogen bond network, we studied both the *trans*-membrane passive permeation properties for a set of neutral drugs (Milanetti et al., 2016) and the properties of non-steroidal anti-inflammatory drugs to predict the extraction recovery of NSAIDs from biological fluids set by solid-phase extraction (Milanetti et al., 2019). When amino acids solvation properties are studied, the main limitation of this method relied on considering a single amino acid in solution instead of inserting it in a functional protein chain. Moreover, the method was developed uniquely for the TIP4 water model, limiting its use to most molecular dynamics simulations (Babiaczyk et al., 2010).

Since the characteristics of the neighboring residues influence the hydropathy of the examined amino-acid, in this work we define the hydropathy properties of each amino acid taking into account the structural environment that surrounds it. In this way, we incorporate the effects of the own characteristics of each amino acid, as well as the chemical and structural properties induced by the surrounding environment.

Furthermore, the method has atomic resolution (Leopizzi et al., 2017), meaning that, given a protein, it is possible characterizing not only a single residue or a set of residues, but we can also quantify the hydrophobic and hydrophilic properties of a set of atoms that contribute to the formation of a portion of the molecular surface. This perspective is particularly important for the improvement of predictive methods of protein-protein interactions (Nicolau et al., 2014). In addition, we have also extended the method to other models of water molecules, especially those typically used for molecular dynamics simulations of proteins, enabling the application of our approach also to the trajectories of simulations already performed.

In particular, we have selected a representative set of experimentally solved protein structures and for each of them, we performed an extensive molecular dynamics simulation. We thus studied the hydropathy profile of the amino acid when they are in different protein structural environments, underlining that, especially for some residues, the solvation properties can sensibly differ according to the characteristics of the different neighborhoods. The analysis of our results allows us to define different regions in a plane describing the hydrophobicity and hydrophilicity properties: each residue belonging to the proteins in our dataset is a point on this plane and its position is not only due to its own chemical properties but also to the nature of the residues closest in structure.

The goodness of the characterization proposed here was evaluated considering the average positions of the residues on the two planes, classifying them by amino acids. These results are in perfect agreement with the hydrophobicity measurement of a biological experimental scale, which is considered the state of the art in this field (Hessa et al., 2005). Furthermore, the dispersion of the residue set for each amino acid was analyzed to underline how the nature of the residues belonging to the structural neighborhood has an important effect on the single residue characterization.

## 2 Results and Discussion

### 2.1 Hydropathy Profile for Single Residue in a Specific Protein Environment

In this section we explain the idea we adopted for the calculation of the amino acid solvation properties, studying the distance and the orientation of water molecules with respect to a solute molecule. We investigated the hydropathy of residues in their natural environment, i.e. inserted in a functional and folded protein chain.

**FIGURE 1:**
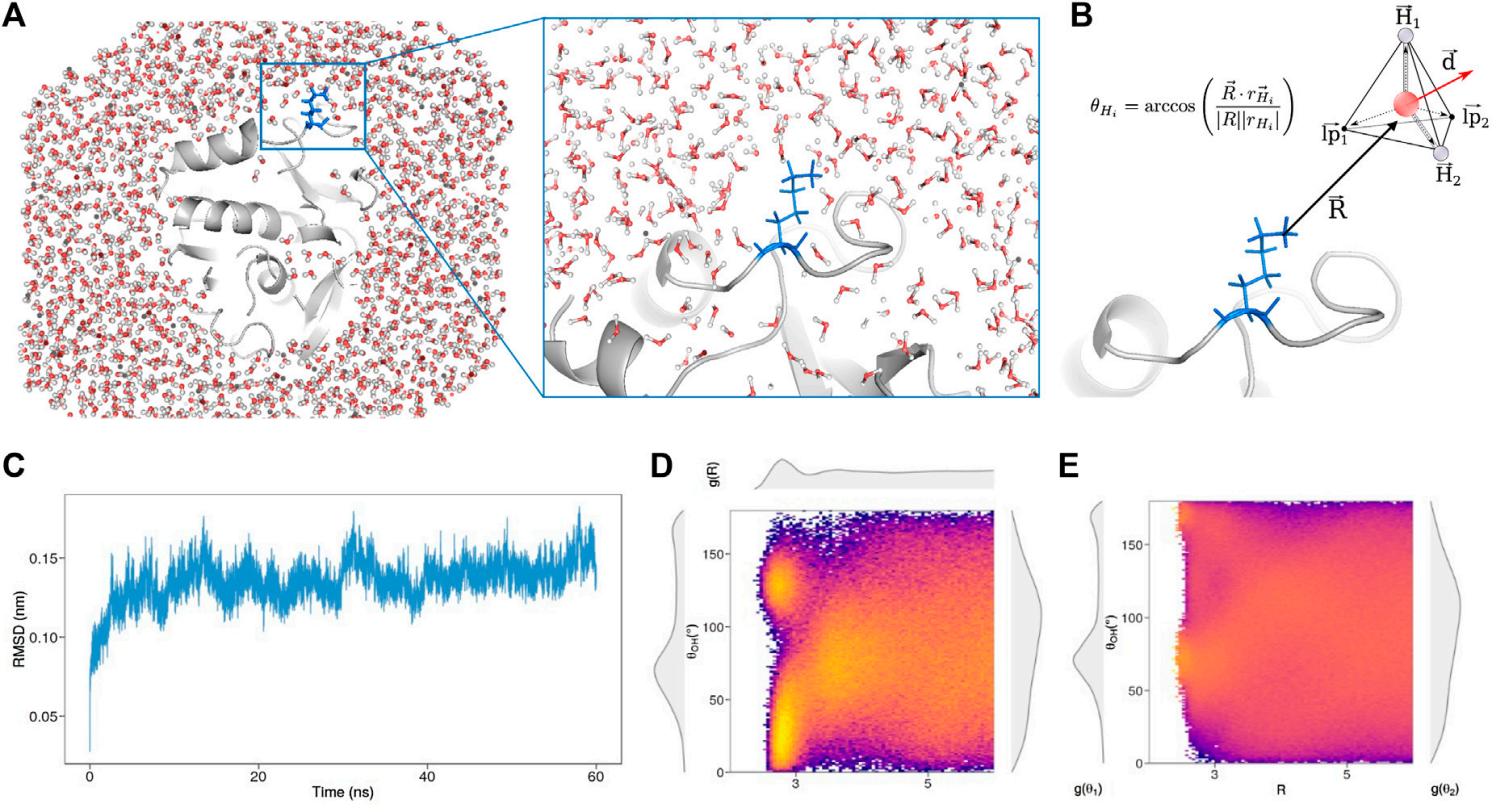
**(A)** Snapshot taken from the molecular dynamics simulation of Concanavalin B (PDB id: 1CNV) performed with explicit solvent. The protein structure is represented in grey, while blue sticks (also zoomed on the right) highlight the position and orientation of an explicate residue, Lys 258, with respect to the surrounding water molecules **(B)** The disposition of each water molecule around a given residue is described representing each solvent molecule as a tetrahedron and evaluating the angles, θ, formed by each vertex of the tetrahedron with the vector, $\vec{R}$ that joins the nearest heavy atom of the residue with the water oxygen atom. The oxygen atom is used as both center of the tetrahedron and origin for the angle definition. The water dipole, $\vec{d}$ is depicted as a red arrow **(C)** Root mean square deviation (RMSD) of the protein-heavy atoms as a function of time, using the initial structure as a reference **(D)** Joined probability distribution, $P_{j}\left(R,\theta \right)$, of finding a water molecule at distance *R* from the residue with one angle, θ of the corresponding tetrahedron. Top, left and right side plots show the marginal probabilities, P(R), $P_{j}\left(\theta_{1}\right)$, and $P_{j}\left(\theta_{2}\right)$, respectively **(E)** Conditional probability distribution, $P_{c}\left(\theta |R\right)$. Left and right side plots show the marginal probabilities, $P_{c}\left(\theta_{1}\right)$, and $P_{c}\left(\theta_{2}\right)$, respectively.

To do so, we selected 20 proteins of known structure from the dataset collected by Hensen *et al.* (Hensen et al., 2012) (see Methods for details), searching very different proteins in terms of structural features to make the analysis as general as possible. In this perspective, we analyzed the SCOP class (Andreeva et al., 2014; Andreeva et al., 2020; of each of the selected protein, demonstrating as our dataset covers several different folds and therefore ensuring the generality of our findings (See Supplementary Table S1). For each of these proteins, a molecular dynamics simulation of 60 ns was performed, studying the behavior of the explicit solvent molecules around the solute (Figure 1A), after the equilibration time (Figure 1C). To testify that we sampled configuration only after the equilibration in all the simulations we performed, we reported in Supporting Information the Root Mean Square Deviation and the Solvent Accessible surface as a function of time for all the proteins (See Supplementary Figures S1–S2).

We note that the explored time span allows us to well grasp the organization of surface waters, while much longer simulations would be needed to consider also the effect of structural water molecules.

According to our method, each solvent molecule can be schematized as a tetrahedron, with the water oxygen in the center and the vertices constituted by the two hydrogen atoms and the two lone pair electrons (Figure 1B), so as each water molecule can form up to four hydrogen bonds (HB). In particular, we associate any water molecule to the closest atoms of the solute focusing only on the first hydration shells, i.e. water molecules closer to any solute atoms than 6 *Å*. Since each water molecule is assigned to one solute atom, for each water molecule the solvent behavior is represented by three quantities representing the position and the orientation with respect to the solute: the distance *R* between the oxygen atom and the closest heavy atom of the solute, the *hydrogen bond angle* θ and the *dipole angle* ϕ. Each hydrogen bond angle is defined as the angle formed between the *R* and each vertex of the tetrahedron using the oxygen atom as the origin. Similarly, the dipole angle is built using the vector *R* and the dipole moment $\vec{d}$ (see Figure 1B for a sketch). In this work, we focus on *R* and θ, since these quantities allow a complete characterization of the solute hydropathy. Indeed, a non-polar (hydrophobic) molecule in an aqueous solution interacts with the solvent only through van der Waals forces. Since the Coulombic interaction among $H_{2}O\text{s}$ is strong, water molecules privilege their internal HBs contacts. Alternatively, the interplay between polar or charged molecules and solvent occurs mainly via Coulombic forces, attracting one of the hydrogens or one of the lone pair electrons toward the solvent atom. Therefore, when a hydrophobic solute is examined, water molecules place one of the faces of the tetrahedron toward the solute in order to leave all possible HBs available; on the contrary, a water molecule close to a polar or a charged solute reorients itself to point toward him one of its lone pairs or hydrogens.

In a nutshell, given the set of atoms composing an amino acid, we carry out statistical analysis of the orientations of the water molecules that hydrate them. In Figure 1D we show a colormap reporting the joint probability to observe a water molecule with a given *R* and θ in the surroundings of the Lys 258 belonging to Concanavalin B (PDB id: 1CNV). As we can see also from the marginal distributions on the panel sides, well-defined peaks reflect the solvation properties of the residue in the protein environment.

On top of Figure 1D, we report P(R), the probability density distribution of finding a water molecule at a distance *R* from the solute, where *j* is the subscript indicating that the probability density is extracted from the joint probability. The curve is characterized by two maxima (this happens for almost all the amino acids), and it is, therefore, possible to identify the first and the second shell of hydration, after which there is the bulk water. On the right and left part of Figure 1D we show $P_{j}\left(\theta_{1}\right)$ and $P_{j}\left(\theta_{2}\right)$, the probability density distribution of finding a water molecule with a certain HB orientation in first or second shell respectively, that is having a *R* in the shells defining interval (see Methods).

It has been demonstrated that, in order to improve the resolution of the description of first and second solvation shells and to achieve a better characterization of the solute features, the adoption of the *conditional probabiliy* represent a powerful tool (Babiaczyk et al., 2010). Indeed in this formalism, we report the probability of having a certain θ, conditional on the solvent locating at a distance R from the solute atom (See Methods for further details). Figure 1E shows the colormap of the conditional probabilities related to Lys 258 and the corresponding probability densities will be indicated with the subscript *c*.

### 2.2 Joint and Conditional Probability for Residue Characterization

**FIGURE 2:**
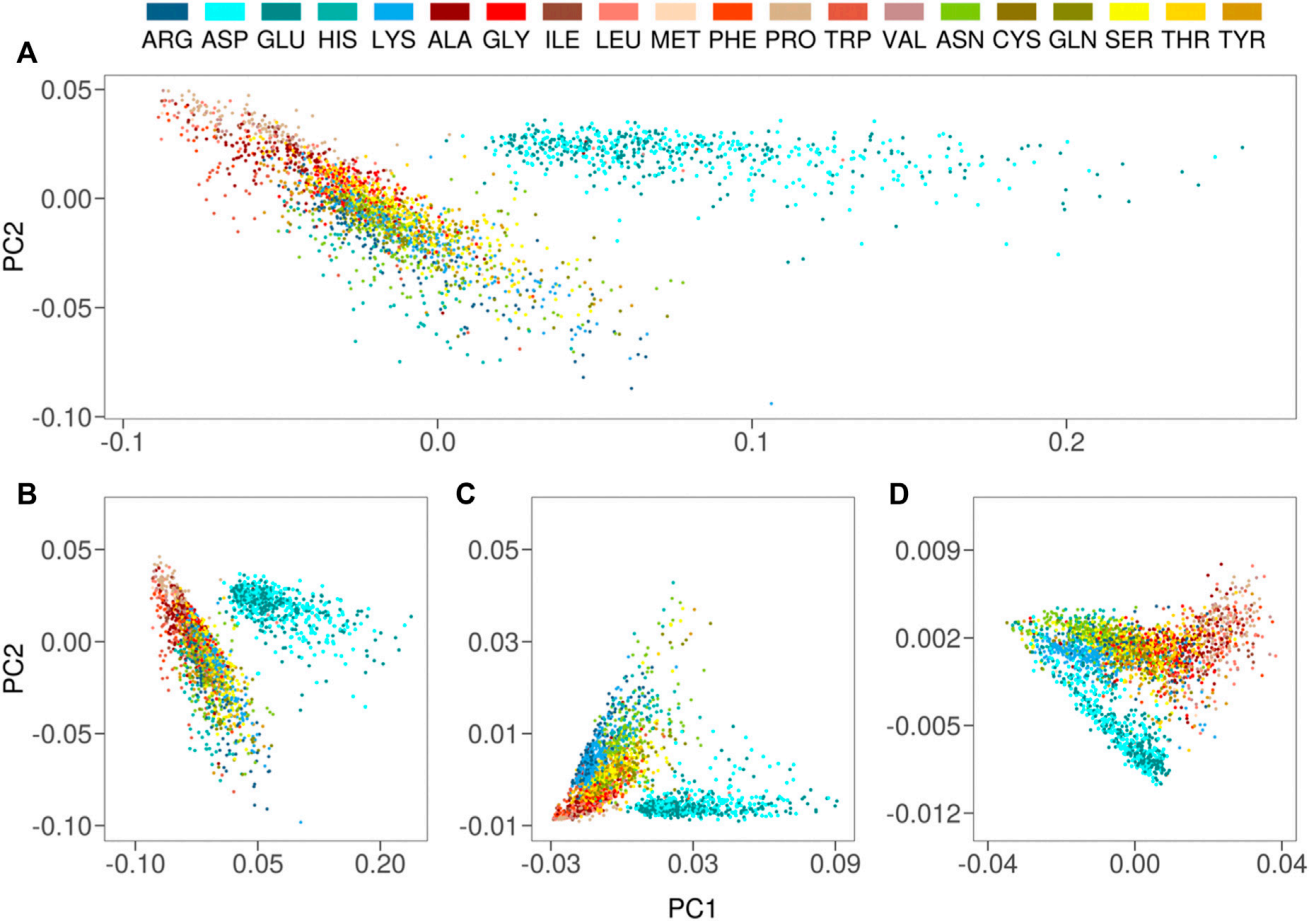
**(A)** Projection along the first two principal components of the residues in the Protein dataset as obtained by a PCA analysis using P(R), $P_{j}\left(\theta_{1}\right)$, and $P_{j}\left(\theta_{2}\right)$ as descriptors for each residue. Each dot in the plane represents a residue, with different colors corresponding to different amino acids **(B)** Same as **A)** but using only the P(R)s as descriptors for each residue **(C)** Same as **A)** but using only the $P_{j}\left(\theta_{1}\right)\text{s}$ as descriptors for each residue **(D)** Same as **A)** but using only the $P_{j}\left(\theta_{2}\right)\text{s}$ as descriptors for each residue.

For each solvent-exposed residue in our dataset, we built an hydropathy profile juxtaposing their P(R), $P_{j}\left(\theta_{1}\right)$ and $P_{j}\left(\theta_{2}\right)$. In this way, each residue is statically characterized by the positions and the orientations of the water molecules surrounding it during the simulation. We obtained a very interesting separation of the amino acid hydropathy by applying a Principal Component Analysis (PCA), where the system is rotated to go into the reference system which maximizes the variance of the data. In Figure 2A, we show the two principal components (percentage of explained variance equal to 88%): each point in this plot represents a given residue explored in its protein environment at physiological pH, and the 20 natural amino acids are colored differently. In particular, charged residues are colored in shades of blue, the non-charged polar residues in red while the hydrophobic residues are depicted in shades of yellow. Interestingly, PCA analysis reveals that residues with similar features are clearly grouped together. In particular, the negatively charged residues, Glu and Asp, form an isolated group, underlining their peculiar behavior in solvent interaction, while in the main cluster of residues each region is characterized by a preference for a certain type of residues.

We also performed a PCA analysis considering separately P(R), $P_{j}\left(\theta_{1}\right)$ and $P_{j}\left(\theta_{2}\right)$. Results are reported in Figures 2B–D respectively. We can notice that the two PCA analyses gave very similar results. According to us, this could mean that, when the joint probability is used to build the profile, the dominating signal is related to the water molecules position, while the information about its orientation gets mainly overwhelmed.

To obtain a finer representation of the all water molecule “signals”, we decided to use the conditional probability to amplify the angular aspect of the hydropathy profile.

**FIGURE 3:**
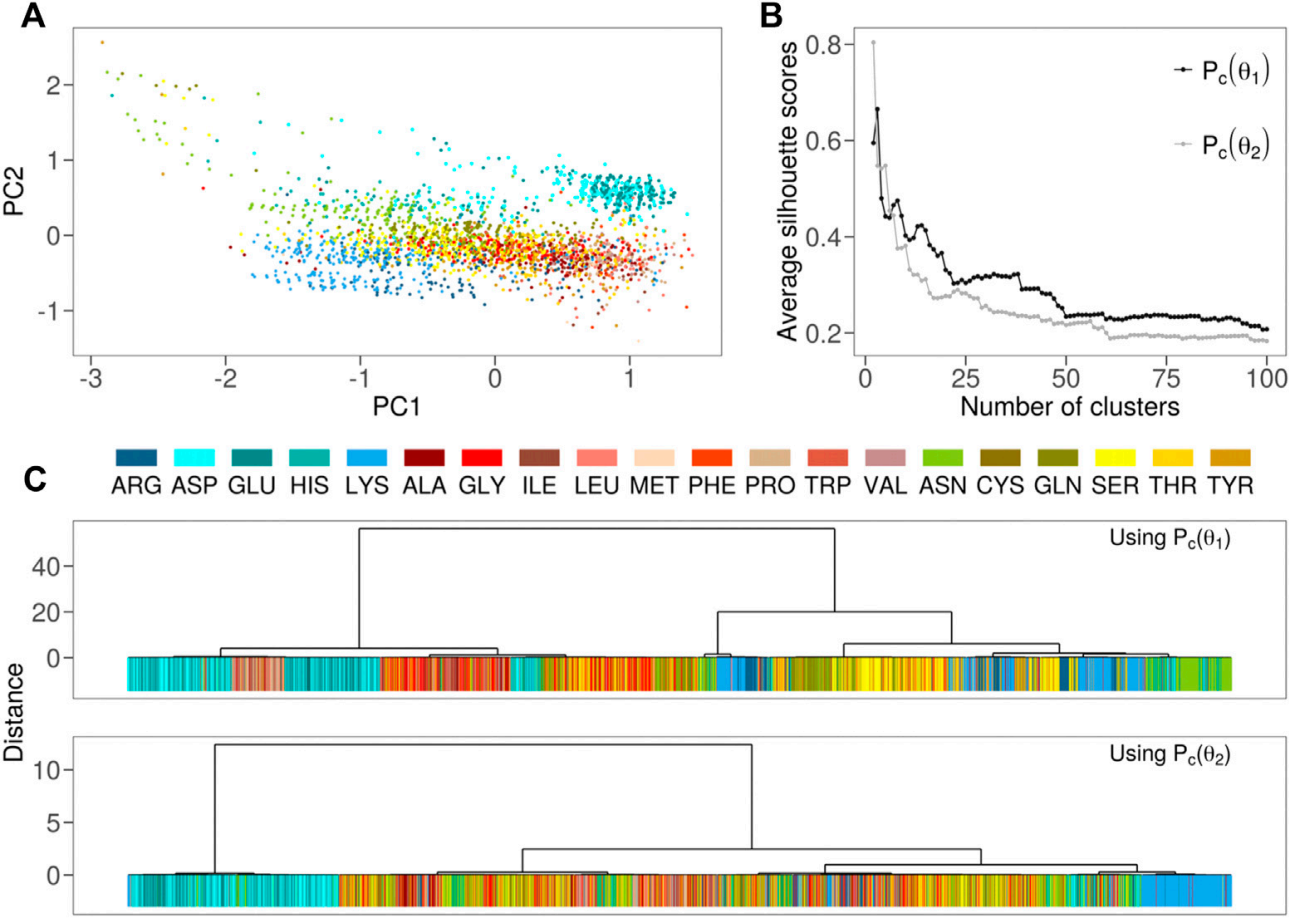
**(A)** Projection along the first two principal components of the residues in the Protein dataset as obtained by a PCA analysis using P(R), $P_{c}\left(\theta_{1}\right)$, and $P_{c}\left(\theta_{2}\right)$ as descriptors for each residue. Each dot in the plane represents a residue, with different colors corresponding to different amino acids **(B)** Cluster of the residue forming the Protein dataset using the $P_{c}\left(\theta_{1}\right)$ **(top)** or $P_{c}\left(\theta_{2}\right)$ **(bottom)** as descriptors for each residue **(C)** Average silhouette score as a function of the number of clusters considered in **(B)**.

To this aim, we performed the same PCA analysis using the $P_{c}\left(\theta_{1}\right)$ and $P_{c}\left(\theta_{2}\right)$ as obtained from the conditional probabilities together with *p*(R). The result is reported in Figure 3A. We can identify four macro-regions: the negatively charged (blue dots) amino acid region (PC1≃1, PC2≃0.8), the positively (cyan dots) charged region ( PC1≃−1, PC2≃−0.5), hydrophobic (red dots) amino acid portion (PC1≃0.8, PC2≃−0.2) and the polar non charged (yellow dots) residue zone (PC1≃−0.2, PC2≃0).

Next, we performed hierarchical clustering of the residues based separately on the two angular density distributions (see Figure 3B). The high values achieved by the silhouette analysis (see Figure 3C) indicate that different subdivisions of residues are possible. For different types of groupings of residues, we note that both $P_{c}\left(\theta_{1}\right)$ and $P_{c}\left(\theta_{2}\right)$ are able to separate amino acids in several clusters composed of amino acids with different biochemical features.

It is worth noting that $P_{c}\left(\theta_{1}\right)$ well isolates a group of hydrophobic (red) residues from the charged residues (both the positively and negatively charged) but this separation is even more clear by using the $P_{c}\left(\theta_{2}\right)$ parameter.

### 2.3 Hydrophobic and Hydrophilic Properties of Amino Acid Side Chains in the Native Structure

**FIGURE 4:**
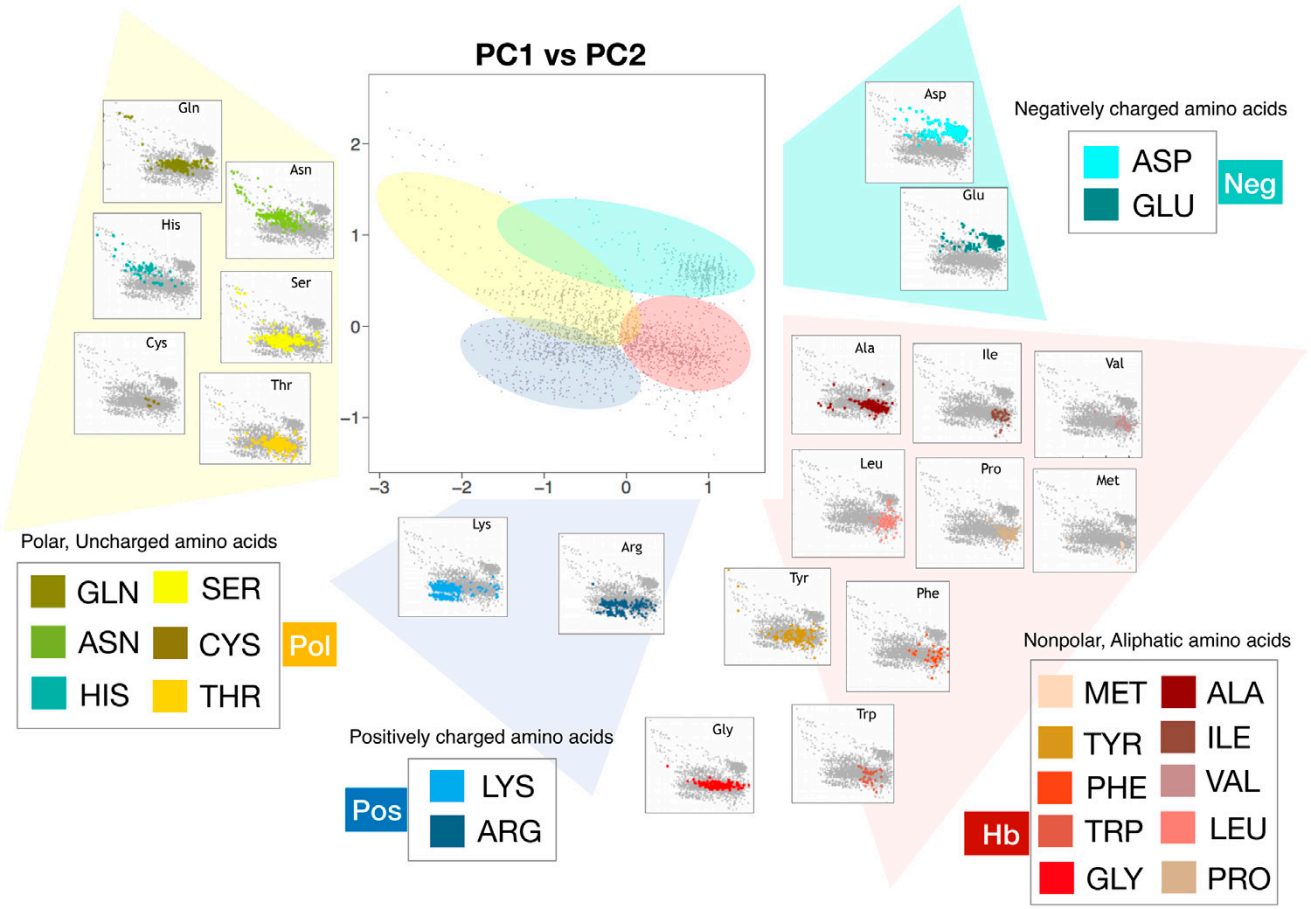
Representations in the plane identified by the first and second principal components of all the residues comprising the 20 proteins of the Protein dataset (grey dots). The PCA analysis has been carried out using for each residue the observed P(R), $P_{c}\left(\theta_{1}\right)$, and $P_{c}\left(\theta_{2}\right)$ computed as described in the Methods. In each panel, dots corresponding to the same kind of amino acid are highlighted with different colors.

The PCA plane we obtained using conditional probabilities (Figure 3A), is a schematic and meaningful description of the solvation properties of the amino acids when they are studied in the native environment. In fact, it is a clever representation of the behavior of the solvent molecules that hydrate protein residues. In Figure 4 we depicted in the PCA plane the points regarding each of the 20 natural amino acids of our dataset using different colors. This way to measure hydropathy characteristics, reporting them as “explored regions” with different chemico-physical features by the amino acid rather than single values assumed by the molecule itself, allowed us to better illustrate the results we obtained. In fact, we demonstrate in this way that some amino acids explore peculiar regions in this plane while other amino acids like Arg, Tyr, Trp, and Thr, clearly populate overlapping regions of the plane. According to us, this may reflect the plasticity of some residues, to emphasize differently hydrophobic or hydrophilic aspects of their atomic structure in different protein local environments due to different biological contests. We summarize this concept of “hydropathy explored regions” in Figure 4 where we defined four portions of the PCA plane according to the kind of residues that explores these areas. We identified the explored hydrophobic area (“Hb” area, depicted in red in Figure 4) in which Ile, Leu, Phe, Val, Pro, and Met residues are very well focused and in good qualitative agreement with previous hydrophobic scales. Then we mapped a clear negative charge explored area (“Neg” area depicted in cyan) where Asp and Glu clusterize. A third portion of PCA plane was defined as positive charge explored area (“Pos” area, depicted in blue in Figure 4) where almost all Lysines of our dataset well converge and Arginine side chain is present for half of the observed configurations; according to us, Lysine explores in few cases the Hb area probably due to the long aliphatic chain, that in some cases outweighs the hydrophilic character.

The presence of Arginine even in the Hb area is biologically very relevant because our result is connecting biological and biophysical principles of Arginine behavior in native proteins: this trend may be impossible to explain by using a just single hydropathy value. In fact, according to us, Arginine hydropathy can vary drastically within a protein environment and so we could define it as a Janus-headed side chain. This observation agrees with experimental data related to this amino acid. In fact, previous experiments by C. Preston Moon and Karen G. Fleming *et al.* (Moon and Fleming, 2011) clearly demonstrated that a membrane protein can accommodate an Arginine side-chain placed near the apolar middle of a lipid bilayer with much less cost in energy than has been previously predicted (Dorairaj and Allen, 2007; MacCallum et al., 2007). In fact, the guanidino group of Arginine could interact with non-polar aromatic and aliphatic side chains above and below the guanidinium plane while hydrogen bonding with polar side chains is restricted to in-plane positions. Related to this point we would like to remember that the first solved structure of a voltage-gated potassium channel (Schow et al., 2011), gave rise to many discussions about the energetics of the interactions between Arginines and lipids, as the structure suggested a gating mechanism in which charged Arginines were exposed to the hydrophobic bilayer interior.

We further observed on the left side of the PCA plane and located between Neg and Pos areas, a region we defined polar explored region (“Pol” area, depicted in yellow in Figure 4) were polar, uncharged amino acids, at physiological pH, are positioned: the location of the area qualitatively agrees with the residue group features of these amino acids that are more hydrophilic than those of the Hb area because they contain functional groups that form hydrogen bonds with water. This class of amino acids includes Ser, Thr, Cys, Asp, and Gln. The presence of this polar area agrees with studies of Peters *et al.* about the assessment of the most accurate hydrophobicity scale (Peters and Elofsson, 2014). They demonstrated that better hydrophobic scales rank the polar amino acids Gln and (in particular) Asn as less hydrophobic. It is interesting to underline that even this polar area overlaps with the Hb area, in agreement with the concept of the ability of amino acids to explore several hydrophilicity-hydrophobicity regions.

**FIGURE 5:**
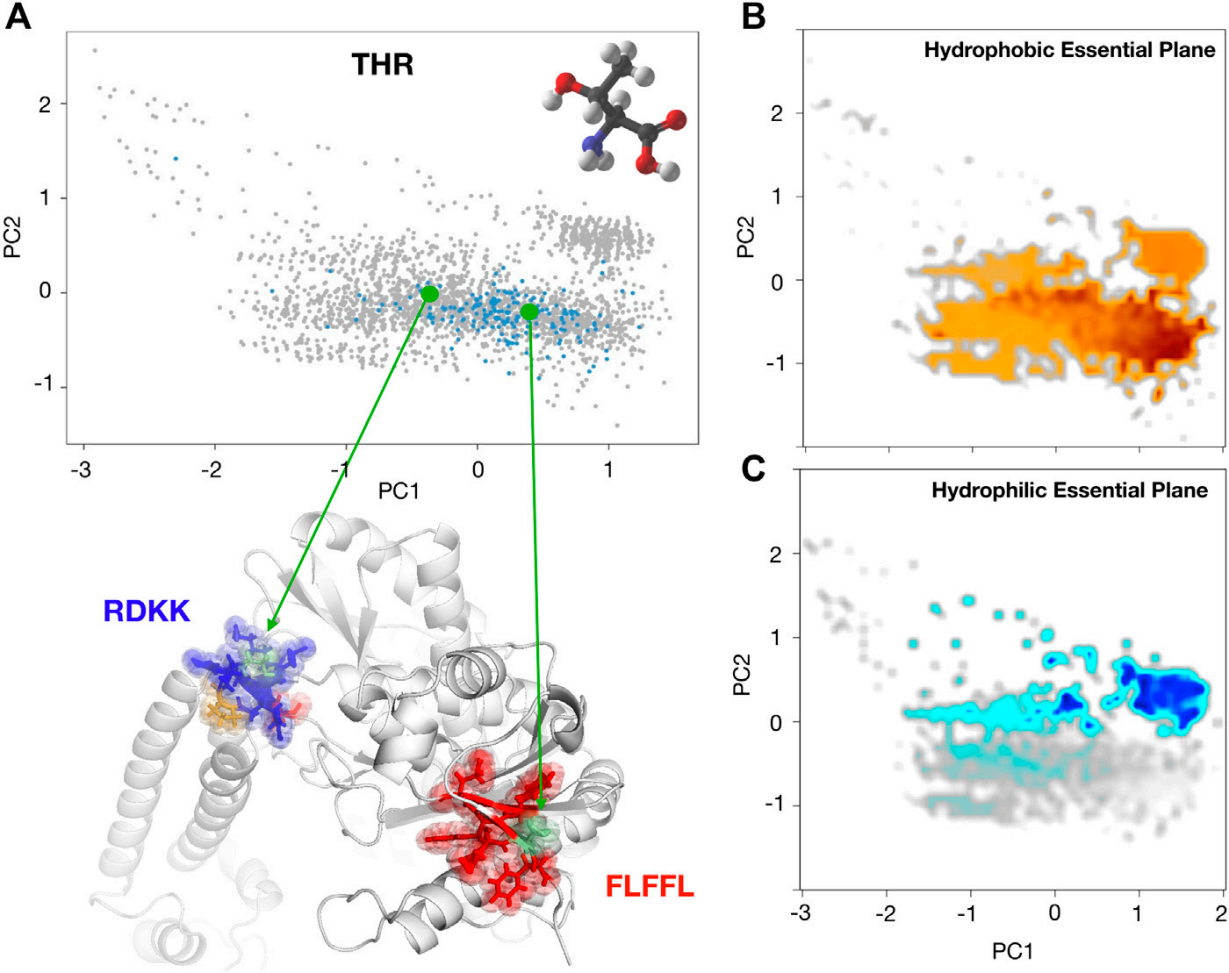
**(A)** Projection along the first two principal components of the residues in the Protein dataset as obtained by a PCA analysis using P(R), $P_{c}\left(\theta_{1}\right)$, and $P_{c}\left(\theta_{2}\right)$ as descriptors for each residue. Each dot in the plane represents a residue. Blue dots highlight the positions in the plane of all Threonine (Thr). Green dots correspond to two cases where the surroundings the considered Threonine is composed of hydrophilic (blue) or hydrophobic (red) amino acids. The average value of the hydrophobicity index **(B)** and of the hydrophobicity index **C)** of the WOPHS scale have been calculated for each element of the grid with which the plan is partitioned.

To better point up this concept, we would like to report the case of the Threonine (Figure 5A) hydropathy analysis in two different contexts. We selected two Threonine residues, Thr 599 and Thr 302 both belonging to the same proteins (PDB:1xwl), characterized by different positions on the PCA plane. The reason for this different behavior in terms of solvent interaction has to be sought in the neighbor residues. In particular, the Thr within the polar region is surrounded by three charged residues (RDKK, reported in blue in the Figure) that inevitably influence his hydrophilic behavior; on the other hand, the Thr within the non-polar zone is enclosed in a set of non-polar residues (FLFFL, in red in the Figure), thus forming an overall hydrophobic region.

Another interesting example is represented by Threonine and Tryptophan. They are straddling the polar and hydrophobic areas and this behavior confirms that our approach is correct. In fact, Tryptophan and Tyrosine can be involved in interactions with ligands that contain aromatic groups via stacking interactions. However, tryptophan has nitrogen in its side chain and Tyrosine has oxygen, allowing hydrogen bonding interactions to be made with other residues or even solvent molecules, commonly seen in polar amino acids like Serine, which has oxygen in its side chain. But we should also keep in mind that Tryptophan has an indole function, but its lone pair of nitrogen is involved in the aromatic system. Thus, it makes only weal H-bonding, which could be not good enough to categorize as “polar”. All these observations are in agreement with the fact that Tyrosine and Triptophane side chains are the typical cases for which numerical values obtained for characterization of the hydrophobicity are controversial, being identified as hydrophobic in some studies (Levitt, 1976; Sweet and Eisenberg, 1983) but hydrophilic in others (Ooi et al., 1987; Oobatake and Ooi, 1988) and our concept of “explored region” should be the right approach.

At the end of this qualitative analysis, we decided to support our speculations by introducing also quantitative data relative to the side chain hydropathy characterization in the native protein context. Although it was not our aim, as proof of the significance of our hydrophobicity/hydrophilicity representation, we developed a mean hydrophobicity measure for each residue $\left(H_{r}\right)$ (see methods for details). We achieved a very good agreement with the biological hydrophobicity scale (or the Hessa scale), which is based on *in vitro* experiments where the recognition of artificial helices by the Sec translocon was measured (Hessa et al., 2005). However, it can be noted that, in this case, the local microenvironment is not known. For example, residues in the helical segment might be interacting with other parts of the protein rather than interacting with lipids or water. The insertion by the translocon might also be a non-equilibrium process. In particular, in order to highlight the mean properties obtainable from this plot, we calculated the centroids of the points regarding each of the 20 natural residues. Using as reference the position of Isoleucine, indicated as the most hydrophobic residue here (Hessa et al., 2005), we calculate the radial and the angular distance of each centroid with the Isoleucine centroid (see Methods for details). In this framework, the higher is the distance with Isoleucine higher is the hydrophilicity of the residues. Notably, we found a strong linear correlation (R = 0.84) between the ΔG of amino acids side chains in the translocon scale and their values of mean hydrophobicity, $H_{r}$ of our native-protein scale (Figure 4 and in Table 1). meaning that our solvation analysis greatly reproduces one of the best performing hydrophobicity scales (Peters and Elofsson, 2014).

**TABLE 1:** Results of the analysis of the essential plane shown in Figure 3A. For each amino acid, we report the number of cases in which it is found solvent-exposed in simulation and the percentage with respect to all the solvent exposed residues (Occurrence); the hydrophobicity values we obtained with our geometrical characterization and the gyration radius, a measure of the dispersion of the points regarding each residues.

Res	Occurrence	$H_{r}$	Gyration radius
ALA	148 (5.4%)	0.47	0.57
ARG	168 (6.1%)	1.60	0.64
ASN	237 (8.6%)	2.03	0.68
ASP	300 (10.9%)	3.20	0.59
CYS	7 (0.3%)	0.59	0.20
GLN	205 (7.5%)	1.26	0.67
GLU	266 (9.7%)	3.32	0.55
GLY	166 (6.1%)	0.80	0.52
HIS	60 (2.2%)	2.29	0.76
ILE	38 (1.4%)	0.00	0.33
LEU	59 (2.2%)	0.79	0.41
LYS	268 (10.4%)	2.76	0.61
MET	12 (0.4%)	0.38	0.76
PHE	34 (1.2%)	0.24	0.52
PRO	97 (3.5%)	0.55	0.27
SER	254 (9.3%)	1.46	0.67
THR	197 (7.2%)	0.66	0.53
TRP	38 (1.4%)	0.65	0.37
TYR	129 (4.7%)	0.83	0.77
VAL	39 (1.4%)	0.46	0.35

Indeed, it is interesting to note that even if the mean properties of the 20 residues can be successfully described using this representation, looking at the plots in Figure 4 it emerges clearly that points belonging to the same amino acid category can spread a lot on this plane, meaning that even the same amino acid can be characterized by very different hydropathy when it is inserted in different environments. Quantitatively, as a measure of the dispersion of the points regarding the various residues, we calculated the amino acid gyration radius (see Methods). We report the results in Table 1.

It results that residues with a well known hydrophobic tendency, such as Proline, Isoleucine, Valine, experience a low variability since they repel water very strongly. On the other hand, residues with a less defined solvent preference, such as Asparagine, Tyrosine, Methionine, are characterized by higher gyration radius values, meaning that they can modify their features influenced by the surroundings.

In light of all these considerations, using the hydrophobicity and hydrophilicity scales presented here (Bonella et al., 2014), we built two maps of these characteristics on the conditional probabilities PCA plane reported in Figure 3A. In particular, by placing a square grid on it we can collect all the points inside each square pixel: since each of these points represents a residue with its hydrophobicity and hydrophobicity values, we can mediate these values obtaining a colormap with the hydrophobicity and hydrophilicity observed in that region of the plane. After a smoothing procedure, we obtain the maps depicted in Figures 5B,C. From this perspective, the evaluation of the hydropathy properties of a given amino acid, located in a specific protein sequence and structure, depends on the position it assumes on this plane, and this position surely depends on their own chemico-physical features but also on the characteristics of its structural neighborhood.

An additional analysis showing the correlation between the secondary structure of a residue and its hydration properties is reported in Supporting Information (See Supplementary Figures S3–S5). Using DSSP Touw et al. (2015); Kabsch and Sander. (1983) we labeled each residue with its secondary structure and we evaluated how the different secondary structures are located in the plane reported in Figure 4. It is worth noting that some non-polar residue, such as ALA and LEU, are usually characterized by a low value of the Hydrophobicity index, but when they are found in loops they can exhibit even high value of the index, probably because of the usual high solvent exposure of this secondary structure.

## 3 Conclusions

Investigating the properties of the hydrogen bond network at the interface between hydration water molecules and solute plays a crucial role in the characterization of the physico-chemical properties of the latter. Here, we presented a completely *in-silico* method capable of analyzing the positions and the orientations of water molecules around any residue of protein structures. This allows us to emphasize the contribution to the solvation properties caused by the local structural environment, underlining that not only the nature of single amino acid determines its hydropathy features, but also the types of residues close to it.

In particular, we analyzed the motion of the water molecules belonging to the first two hydration shells for a set of proteins, defining a new description of both the hydrophilicity and hydrophobicity properties. Studying the probability of water molecule’s orientation conditional to the distance to the solute, we built an essential plane of hydrophilicity and hydrophobicity, through a dimensionality reduction of the probability density distribution. On average, the location of each amino acid on this plane is in perfect agreement with its biochemical properties, in fact, an index defined considering the average position of each amino acid has an excellent correlation with one of the state-of-art hydrophobicity scales.

This notwithstanding, the dispersion of each amino acid (considering all the occurrences of a given residue in the proteins of our dataset) is a good marker of its variability in terms of solvation features. Indeed, this dispersion well classifies amino acids with marked properties, such as strong, from amino acids with less pronounced or intermediate hydropathy properties, meaning that the local structural environment in these cases plays a predominant role in modifying their interaction with the solvent.

## 4 Materials and Methods

### 4.1 Protein Dataset and Residue Selection

We consider the dataset proposed by Hensen *et al.* (Hensen et al., 2012), where a collection of 112 representative proteins for each family were reported. From this initial set, we selected the 20 proteins, having 1) longer sequences and 2) no missing or incomplete residues. Considering all proteins together, we ended up with a total of 6,745 residues. For each protein, a molecular dynamics simulation with explicit solvent was performed. Since we were interested in characterizing solvation-related features, we consider only residues found in interaction with more than 50,000 water molecules during the whole analyzed simulation. An interaction between a residue and a water molecule is established if the distance between the oxygen atom of the water and any of the residue heavy atom is smaller than 6 *Å*. We ended up with 2,775 residues.

### 4.2 Molecular Dynamics Simulation

The following protocol was used for each of the 20 simulations. We used Gromacs 2020 (Spoel et al., 2005) and built the system topology using the CHARMM-27 force field (Brooks et al., 2009). The protein was placed in a dodecahedric simulative box, with periodic boundary conditions, filled with TIP3P water molecules (Jorgensen et al., 1983). We checked that each atom of the protein was at least at a distance of 1.1 nm from the box borders. The system was then minimized with the steepest descent algorithm. Next, a relaxation of water molecules and thermalization of the system was run in NVT and NPT environments each for 0.1 ns at 2 fs time-step. The temperature was kept constant at 300 K with v-rescale algorithm (Bussi et al., 2007); the final pressure was fixed at 1 bar with the Parrinello-Rahman algorithm (Parrinello and Rahman, 1980). LINCS algorithm Hess et al. (1997) was used to constraint h-bonds. A cut-off of 12 Å was imposed for the evaluation of short-range non-bonded interactions and the Particle Mesh Ewald method Cheatham et al. (1995) for the long-range electrostatic interactions. Finally, we performed 60 ns of molecular dynamics with a time step of 2 fs, saving configurations every 2 ps. We considered the last 20 ns (10,000 frames) for the analyzes.

### 4.3 Evaluation of Solvent-Residue Geometrical Descriptors

Molecular dynamics simulation data were used to characterize the geometrical disposition of the water molecules around protein residues. In particular, for each protein of the Protein dataset, we sampled 10,000 configurations (one each 2 ps) from the corresponding molecular dynamics simulation. For every water molecule in each frame, we evaluate the minimum distance, *R*, between the water oxygen and the heavy atoms of each protein residue.

Solvent molecules whose oxygen atom had a distance bigger than 6 Å were discarded from all subsequent analyses. All remaining water molecules were then assigned to their nearer residue, again on the basis of the distance *R*.

Then, for each water molecule, we build the tetrahedron having the oxygen atom as the center and the two hydrogen atoms occupying two of the four vertexes. In this way, we ensure that the tetrahedron is always well defined. We indicate with ${\vec{r}}_{H_{1}}$ and ${\vec{r}}_{H_{2}}$ the vectors originating in the tetrahedron center and pointing to the hydrogen atoms; while we refer to the vectors linking the center with the other to vertex as ${\vec{r}}_{{\text{lp}}_{1}}$ and ${\vec{r}}_{{\text{lp}}_{2}}$ (where lp stands for *lone pairs*). Finally, we define also the vector joining the nearest heavy atom of the residue with the oxygen atom of the water molecule, $\vec{R}$, and the dipole moment vector, $\vec{d}$ (see Figure 1 for a sketch).

Once we know the set of six vectors $[\vec{R},\vec{d},{\vec{r}}_{H_{1}}$, ${\vec{r}}_{H_{2}},{\vec{r}}_{{\text{lp}}_{1}},{\vec{r}}_{{\text{lp}}_{2}}]$, we can compute the five angles that efficiently summerize the disposition of the water molecule with respect to the protein residue. In particular,

$$\theta_{H_{i}}=\arccos\left(\frac{\vec{R}\cdot {\vec{r}}_{H_{i}}}{\left|R\right|\left|r_{H_{i}}\right|}\right),$$ (1)

and

$$\theta_{lp_{i}}=\arccos\left(\frac{\vec{R}\cdot {\vec{r}}_{lp_{i}}}{\left|R\right|\left|r_{lp_{i}}\right|}\right),$$ (2)

with i=1,2 identify the orientation of the tetrahedron vertexes with respect to the direction identified by $\vec{R}$, while

$$\phi =\arccos\left(\frac{\vec{R}\cdot \vec{d}}{\left|R\right|\left|d\right|}\right),$$ (3)

measures the orientation of the water dipole.

### 4.4 Joint and Conditional Probability

For each of the 2,775 residues, we computed the hydrogen joint probability, P(R,θ), which gives the probability of finding a water molecule with a given $\theta_{OH-lp}=\theta$ angle at distance *R* from the nearest heavy atom of the reside and dipole joint probability, P(R,ϕ), of finding a water molecule with a given ϕ angle at distance *R*. In both cases, the probabilities are computed discretizing the distance range 0–6 *Å* with steps of 0.05 Å, and the angular interval $0-180^{\circ }$ with a step of $1^{\circ }$. See Figure 1 for an example.

From the joint probabilities, we obtained the distance marginal probability, P(R) as

P(R)=∫dθP(R,θ), (4)

while we calculated the conditional probabilities as

$$P\left(\theta |R\right)=\frac{P\left(R,\theta \right)}{P\left(R\right)}.$$ (5)

Considering each residue as a reference, P(R) encodes the overall probability of finding a water molecule at distance *R* from the reference. As one can see from Figure 1, the typical shape of probability is that of a damped sinusoidal function, showing a series of maxima (and minima) with decreasing amplitude. This behavior originates from the molecular interactions between the residue and the water molecules and those between water molecules. Water molecules tend to form a shell around the residue with a higher density of molecules in correspondence of the P(R) maxima and a lower density in its minima.

Using the P(R) profile, we identified the shells as follows:

- the first shell starts at $R_{0}$ the first non-null value of P(R);
- the border between the first shell and the second $\left(R_{1}\right)$ coincide with the minimum following the maximum in the first shell;
- the end of the second shell, $R_{2}$ coincides with the minimum following the maximum in the second shell.

When the P(R) profile does not allow us to identify the minima, we add them according to their average values calculated on the respective residue.

Once the shells were identified, we calculated $P\left(\theta_{1}\right)$ and $P\left(\theta_{2}\right)$ as

$$P\left(\theta_{i}\right)=\int_{R_{i-1}}^{R_{i}}dRP\left(\theta |R\right),$$ (6)

where i=1,2 and θ can be either the hydrogen angle or the dipole one. The P(θ) were calculated on both the joint $\left(P_{j}\left(\theta_{1,2}\right)\right)$ and conditional $\left(P_{j}\left(\theta_{1,2}\right)\right)$ probability. Since some P(R) exhibited an anomalous profile they were discarded from subsequent analyzes, reducing the dataset to 2,740 residuals. Ultimately, we obtained three descriptors for the conditional and joint probability histograms of the OH-lp and dipole angles: P(R), $P_{j,c}\left(\theta_{1}\right)$ and $P_{j,c}\left(\theta_{2}\right)$. Analyses were performed using R standard libraries (R Core Team, 2020).

### 4.5 Principal Component Analysis and Clustering

Principal component analysis (PCA) was performed over 1) the vector obtained by concatenating the discretized (75 points) probability distribution P(R) for each of the 2,740 residues; 2) on the vector obtained by concatenating the discretized (180 points) probability distribution $P_{j}\left(\theta_{1}\right)$ for each of the 2,740 residues; 3) on the vector obtained by concatenating $P_{j}\left(\theta_{2}\right)$ of all 2,740 residues and 4) using the vector obtained by concatenating together all the previous probabilities. We used the “prcomp” function of R software (R Core Team, 2020). The same procedure has been repeated also using P(R) and the two conditional probability marginals, $P_{c}\left(\theta_{1}\right)$, and $P_{c}\left(\theta_{2}\right)$.

A clustering analysis was performed on the points on the first two components plane relating to $P_{c}\left(\theta_{1}\right)$ and $P_{c}\left(\theta_{2}\right)$ through a hierarchical clustering, using the Euclidean distance and the Ward method as linkage function (Ward, 1963) via the “hclust” function of the “Stats” package of R (R Core Team, 2020). Finally, we computed the Silhouette for the hierarchical cluster via the R package “cluster” (Maechler et al., 2019).

Finally, to measure the dispersion of the points regarding the various residues in the PCA plane, we calculated the amino acids gyration radius as

$$R_{g}=\sqrt{\frac{1}{N}\sum_{i=1}^{N}\left(r_{i}^{2}\right)},$$ (7)

where $r_{i}$ are the distances between each of the N points and the centroid.

### 4.6 Hydrophobicity Measure in Principal Component Plane

Starting from the plane shown in Figure 3A, we defined a measure of hydrophobicity. We take as reference the point *C*, the centroid of all the Ile points with coordinates PC1 = 0.75 and PC2 = −0.39. For a generic point in the plane, *i*, we calculated the distance $d_{i}$ from *C*. Defining the angle variable α, like the one starting from the *x*-axis in an anticlockwise direction, we thus fixed a reference angle, $\alpha_{ref}=2.8rad$. Now it is possible to define, for a generic point *i* on the plane with distance $d_{i}$ and angle $\alpha_{i}$, the Hydrophobicity index as follows:

$$H_{r}=d_{i}+k\left|\alpha_{i}-\alpha_{ref}\right|,$$ (8)

where k=2.

## Data Availability

The original contributions presented in the study are included in the article/Supplementary Material, further inquiries can be directed to the corresponding authors.
