# Supplementary material for: Characterizing Hydropathy of Amino Acid Side Chain in a Protein Environment by Investigating the Structural Changes of Water Molecules Network
Source: Front Mol Biosci. 2021 Feb 26;8:626837. doi: 10.3389/fmolb.2021.626837 (PMC7954116; doi:10.3389/fmolb.2021.626837)
Supplement: Supplementary file 1 [file datasheet1.pdf]

Supporting Information: Characterizing hydropathy of amino acid side chain in a protein environment by investigating the structural changes of water molecules network.

Lorenzo Di Rienzo*,[1] Mattia Miotto*,[2,1] Leonardo Bò,[1] Giancarlo Ruocco,[1,2] Domenico Raimondo†,[3] and Edoardo Milanetti†[2,1]

[1] *Center for Life Nanoscience, Istituto Italiano di Tecnologia, Viale Regina Elena 291, 00161, Rome, Italy*
[2] *Department of Physics, Sapienza University, Piazzale Aldo Moro 5, 00185, Rome, Italy*
[3] *Department of Molecular Medicine, Sapienza University, Rome 00161, Italy*

Proteins Fold Analysis

Since our findings aim to be as general as possible, the selected proteins have to be a representative subset of the protein characteristics.

In this perspective, we selected as a starting point for our work the dataset employed in Henset et al [1]. Indeed, in that work, the authors, investigating the role of dynamics in protein function, selected a dataset of 112 proteins covering a large fraction of known folds, so as their findings can be considered fold-independent. Since this is also our aim, we chose a subset of this dataset. Indeed, when we picked out 20 from the 112 original proteins, we selected the 20 biggest proteins with no missing heavy atoms in their structure. Analyzing the SCOP [2, 3]class of both our 20 and the original 112 structures in the datasets, we built the following table reporting the percentage of proteins belonging to each Scop class.

As it can be noted, all the different folds are well represented in our dataset, and in the Hensen Dataset as well. We can conclude that the conclusions we drawn in this work are largely independent of the protein folds.

**Scop Class**	Protein Dataset (20 proteins)	Hensen Dataset[a](112 proteins)
$all-\alpha$	3 (15%)	12 (10%)
$all-\beta$	3 (15%)	33 (27%)
α/β	10 (50%)	27 (22%)
$\alpha+\beta$	4 (20%)	30 (25%)

[a]we did not include 10 small proteins

Supporting Table I: Percentage of Protein Dataset and Hensen Dataset proteins belonging to the various SCOP fold class.

* The authors contributed equally to the present work.
† Corresponding authors:
edoardo.milanetti@uniroma1.it, domenico.raimondo@uniroma1.it

Molecular Dynamics Stability

The choice of the simulation time has to balance the trade-off between exhaustive sampling of the configuration space (the wider the better) and the computational cost of full-atomistic molecular dynamics simulations.

In this work, we were interested in characterizing the disposition of the solvent molecules around different kinds of protein residues taking into account the effects of the surrounding (when the latter is as close as possible to a realistic condition).

Thus, we run simulations long enough to have for all the 20 proteins an equilibrium regime in terms of Root Mean Square Deviation with respect to the starting configuration and so to let the protein assume a relaxed conformation. In Supporting Figure 1 we reported the results obtained for each of the 20 simulated proteins, where a red vertical line is located at t = 40 ns when we start sampling the configurations.

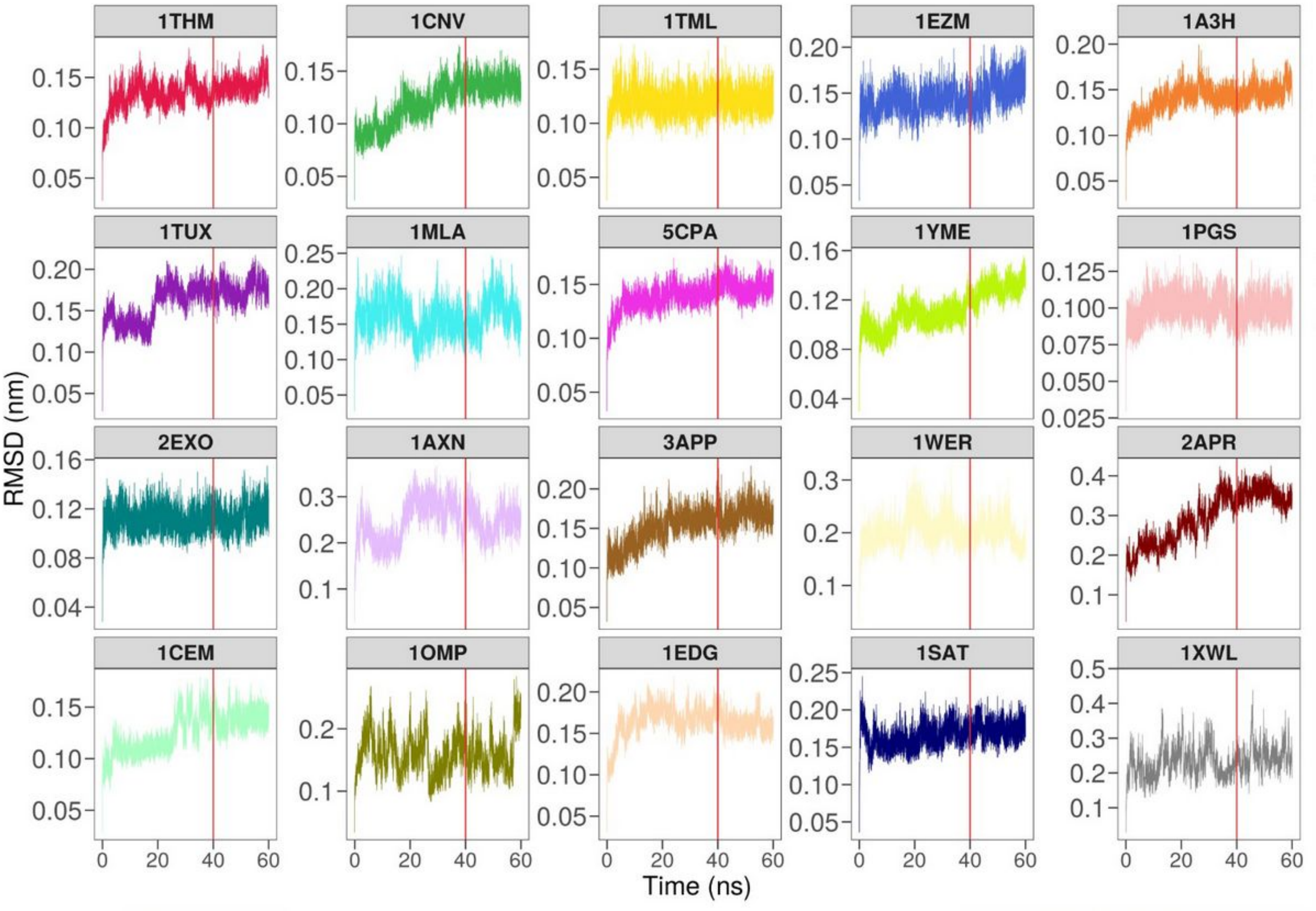

Supporting Figure 1: Root Mean Square Deviation as a function of the time for each of the 20 molecular dynamics we performed. We used as a reference structure the initial configuration.

We thus computed also the Solvent Accessible Surface as a function of the time for each protein dynamics. We reported in Supporting Figure 2 the obtained plots. One can see that most of the variation in terms of SAS takes place in the first 10-20 ns of the trajectory, in correspondence to the most significant changes of the protein structures (see RMSD in Supporting Figure 1). We note that indeed different proteins exhibit different SAS. However, after the initial time span of 20 ns, the solvated area remains quite stable testifying the overall reliability of the used configurations.

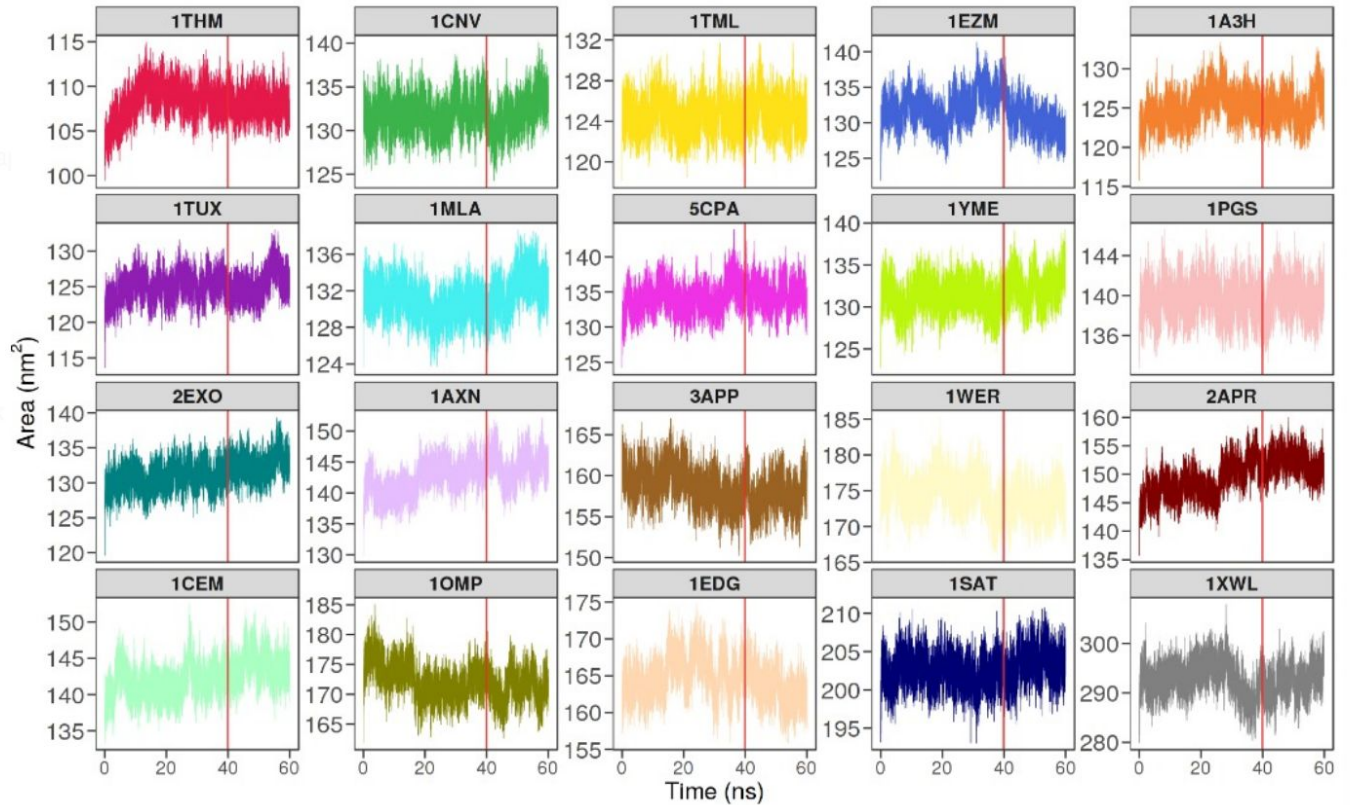

Supporting Figure 2: Protein Solvent Accessible Surface as a function of the time for each of the 20 molecular dynamics we performed.

Secondary Structure Analysis

In order to highlight the effects of local structural organization on the residue hydration behavior, we performed the following analysis about the correlation between residue secondary structure and its hydration features.

In particular, using DSSP [4, 5] we assigned each residue of each of the 20 proteins we simulated to a secondary structure group, according to DSSP categorization. Thus in Supporting Figure 3, we show the projection along the first two principal components of the residues belonging to the 20 proteins in the dataset, as obtained by a PCA analysis employing the conditional probabilities (we report this figure in Fig3.a, 4 and 5.a of the paper).

As explained in the main text, the position of a residue in this plane summarizes its hydropathy behavior. In Supporting Figure 3 we report 8 panels, each showing with a different color the points regarding a given secondary structure, as classified by DSSP. Looking at all the panels, it emerges that the points are not grouped according to their secondary structure category, meaning that the hydration properties of a residue do not depend solely on its secondary structure.

To further investigate this aspect, we investigated how the hydropathy index we defined in the main text correlates with the secondary structure of the residues. In Supporting Figure 4 we reported the distributions of the Hydrophobicity index of the residues belonging to the different secondary structure category, ordered from the more hydrophobic to the more hydrophilic category. On top of the panel, we report the number of residues composing each secondary structure group. It results that the more hydrophobic secondary structure is Hydrogen bonded turn, while residues in isolated β-bridge are characterized by the more hydrophilic behavior (according to the median value), even if these results can be due to the low population of the hydrophilic groups.

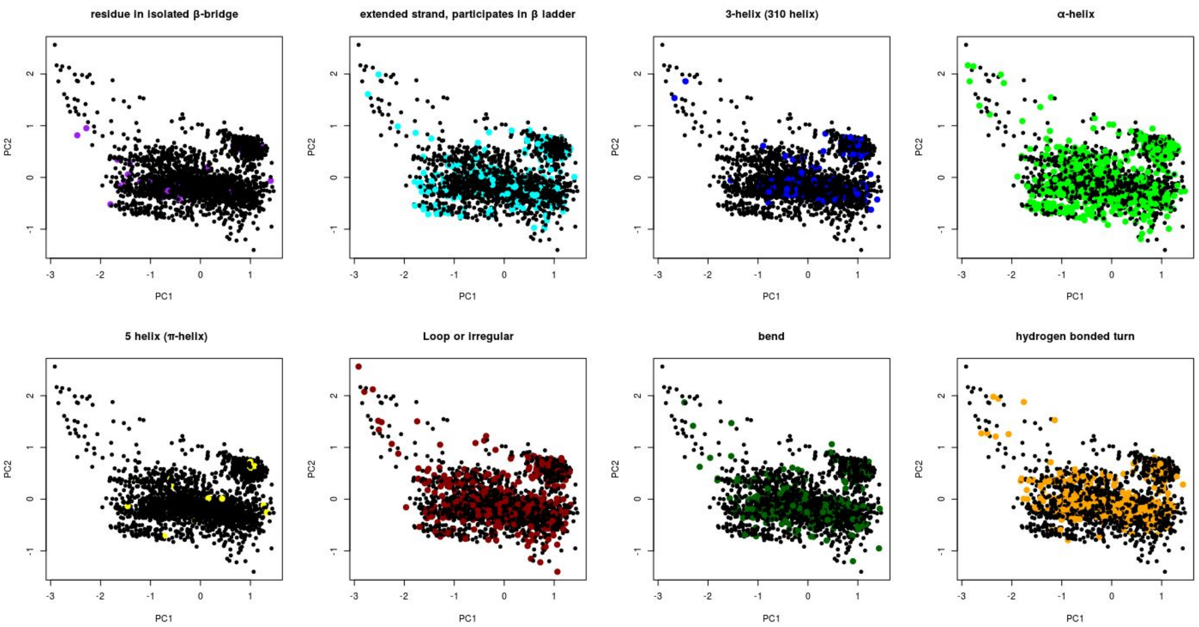

Supporting Figure 3: Projection along the first two principal components of the residues in the Protein dataset as obtained by a PCA analysis using $P(R)$, $P_c(\theta_1)$, and $P_c(\theta_2)$ as descriptors for each residue. Each dot in the plane represents a residue, while in each panel the points corresponding to the different secondary structures are highlighted with different colours.

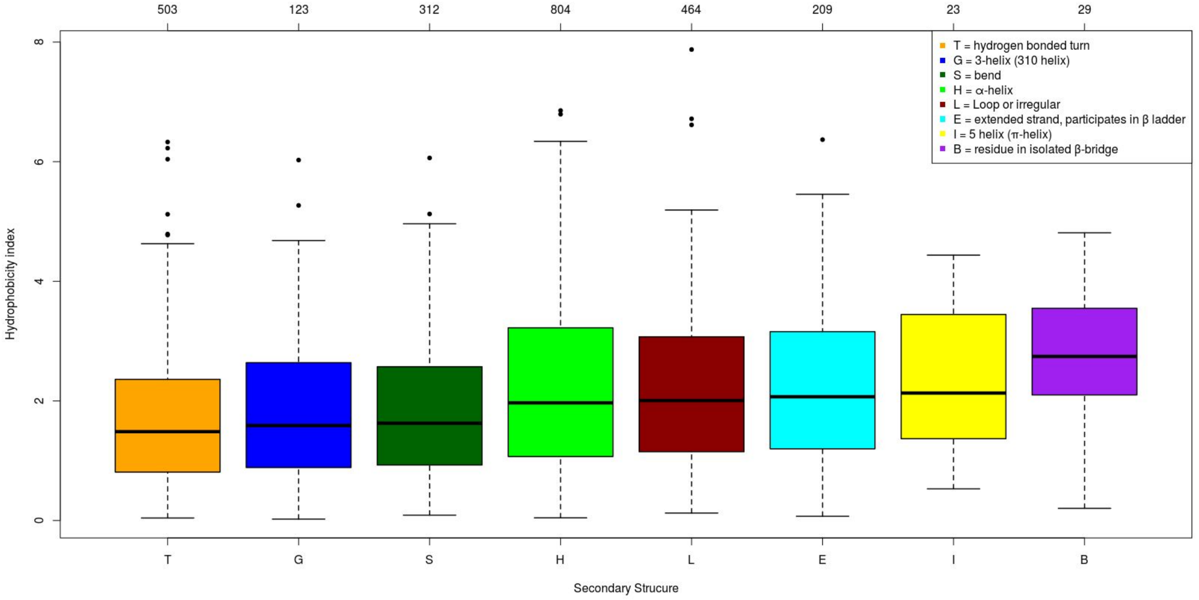

Supporting Figure 4: Boxplot showing the distributions of the Hydrophobicity index of residues grouped according to the secondary structure category they belong. On top of the panel the number of residues composing each secondary structure group are reported.

Interestingly, we then performed this analysis separately for each amino acid reporting the results in the panels of Supporting Figure 5 (The results for CYS, ILE, MET and VAL are not reported because of the low number of studied residues since no secondary structure category have a minimum number of 10 residues). For instance, it is

worth noting the peculiar behavior of ALA and LEU; they are nonpolar amino acid and consequently are usually characterized by a low value of the index, but when they are found in loops (red in the figure) they can exhibit even higher values, probably because of the usual high solvent exposure of this secondary structure.

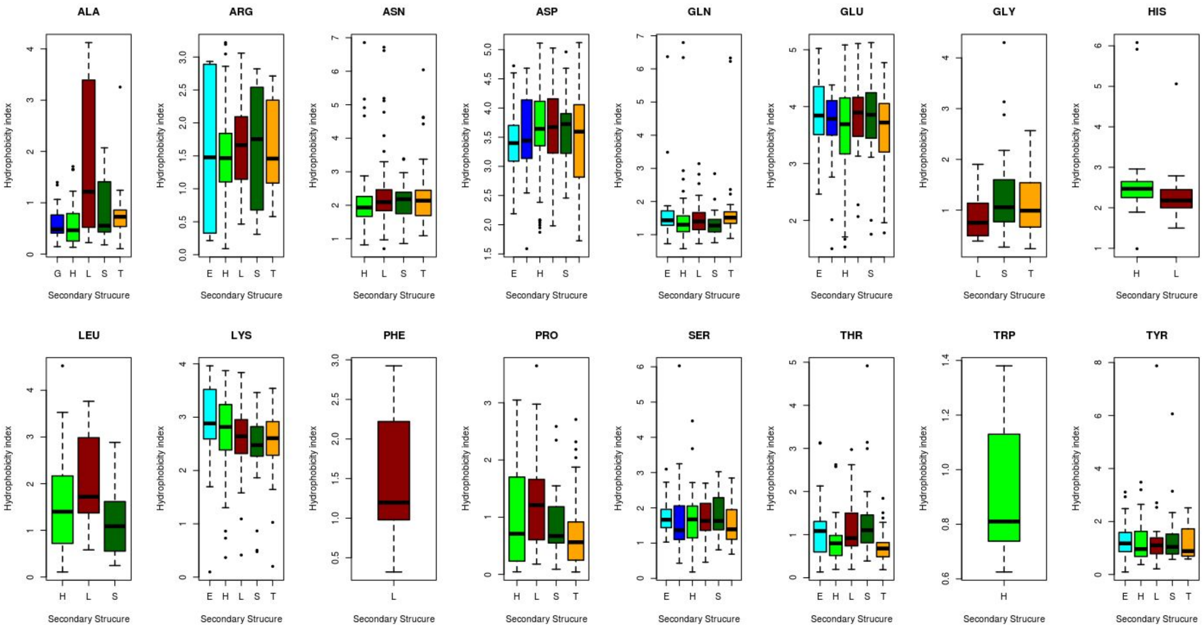

Supporting Figure 5: For each amino acid, the boxplot describes the distributions of the Hydrophobicity index of residues grouped according to the secondary structure category they belong.

[1] U. Hensen, T. Meyer, J. Haas, R. Rex, G. Vriend, and H. Grubmüller, PLoS ONE **7**, e33931 (2012).
[2] A. Andreeva, D. Howorth, C. Chothia, E. Kulesha, and A. G. Murzin, Nucleic acids research **42**, D310 (2014).
[3] A. Andreeva, E. Kulesha, J. Gough, and A. G. Murzin, Nucleic acids research **48**, D376 (2020).
[4] W. G. Touw, C. Baakman, J. Black, T. A. Te Beek, E. Krieger, R. P. Joosten, and G. Vriend, Nucleic acids research **43**, D364 (2015).
[5] W. Kabsch and C. Sander, Biopolymers: Original Research on Biomolecules **22**, 2577 (1983).
